# Long-Lasting Silver Nanoparticles Synthesized with *Tagetes erecta* and Their Antibacterial Activity against *Erwinia amylovora,* a Serious *Rosaceous* Pathogen

**DOI:** 10.3390/plants13070981

**Published:** 2024-03-29

**Authors:** Johana Zarate-Escobedo, Hilda Araceli Zavaleta-Mancera, Ramón Marcos Soto-Hernández, Paulino Pérez-Rodríguez, Alfredo Rafael Vilchis-Nestor, Hilda Victoria Silva-Rojas, Libia Iris Trejo-Téllez

**Affiliations:** 1 Programa de Fisiología Vegetal, Colegio de Postgraduados en Ciencias Agrícolas Campus Montecillo, Montecillo, Texcoco 56264, Estado de México, Mexico; zarate.johana@colpos.mx; 2 Programa de Botánica, Colegio de Postgraduados en Ciencias Agrícolas Campus Montecillo, Montecillo, Texcoco 56264, Estado de México, Mexico; msoto@colpos.mx; 3 Programa de Estadística, Colegio de Postgraduados en Ciencias Agrícolas Campus Montecillo, Montecillo, Texcoco 56264, Estado de México, Mexico; perpdgo@colpos.mx; 4 Centro Conjunto de Investigación en Química Sustentable, UAEM-UNAM, Toluca 50200, Estado de México, Mexico; arvilchisn@uaemex.mx; 5 Programa de Semillas, Colegio de Postgraduados en Ciencias Agrícolas Campus Montecillo, Montecillo, Texcoco 56264, Estado de México, Mexico; hsilva@colpos.mx; 6 Laboratorio de Nutrición Vegetal, Programa de Edafología, Colegio de Postgraduados en Ciencias Agrícolas Campus Montecillo, Montecillo, Texcoco 56264, Estado de México, Mexico; tlibia@colpos.mx

**Keywords:** green synthesis, *Tagetes erecta*, surface plasmon resonance, long-lasting AgNPs, antibacterial activity, *Erwinia amylovora*

## Abstract

A rapid, eco-friendly, and simple method for the synthesis of long-lasting (2 years) silver nanoparticles (AgNPs) is reported using aqueous leaf and petal extracts of *Tagetes erecta* L. The particles were characterized using UV-Visible spectrophotometry and the analytical and crystallographic techniques of transmission electron microscopy (TEM). The longevity of the AgNPs was studied using UV-Vis and high-resolution TEM. The antibacterial activity of the particles against *Erwinia amylovora* was evaluated using the Kirby–Bauer disk diffusion method. The results were analyzed using ANOVA and Tukey’s test (*p* ≤ 0.05). Both the leaf and petal extracts produced AgNPs, but the leaf extract (1 mL) was long-lasting and quasi-spherical (17.64 ± 8.87 nm), with an absorbance of UV-Vis λ_max_ 433 and a crystalline structure (fcc, 111). Phenols, flavonoids, tannins, and terpenoids which are associated with -OH, C=O, and C=C were identified in the extracts and could act as reducing and stabilizing agents. The best antibacterial activity was obtained with a nanoparticle concentration of 50 mg AgNPs L^−1^. The main contribution of the present research is to present a sustainable method for producing nanoparticles which are stable for 2 years and with antibacterial activity against *E. amylovora*, one of most threatening pathogens to pear and apple productions.

## 1. Introduction

There has been growing interest in the green synthesis of silver nanoparticles (AgNPs) because it is considered an eco-friendly, simple, and economic method compared to chemical methods that use toxic compounds [1]. The synthesis of AgNPs mediated by plant extracts has received special attention. These plants are particularly noteworthy due to their richness in bioactive compounds, which facilitate the reduction of Ag^+1^ and the stabilization of the NPs [2,3,4]. Extracts of several plant organs have been used for the synthesis of AgNPs, including the flowers of *Datura inoxia* [5], *Nyctanthes arbor-tristis* [6], and *Tagetes erecta* [7]; the leaves of *Erigeron bonariensis* [3]; the stems of *Caesalpinia pulcherrima* [8]; the fruit of *Prunus armeniaca* [9]; the bark of *Senna alata* [10]; the roots of *Pelargonium sidoides* [11] and *Codonopsis pilosula* [12]; and the seeds of *Jatropha curcas* [13]. Although there is sufficient information on the green synthesis of AgNPs, no studies have focused on the stability and longevity of these particles. The stability of AgNPs over time is very important for maintaining their physical and chemical properties [14,15,16].

*Tagetes erecta* L. (Asteraceae), also known as “cempoalxóchitl” in Nahuatl, is a species native to Mexico and South America [17]. It is cultivated for medicinal, ceremonial, and decorative purposes. *T. erecta* contains several bioactive and antioxidant compounds, such as carotenoids, phenols, flavonoids, α-tocopherol, terpenes, sterols, and tannins [18,19], which contribute to a wide range of applications for developing pesticides, such as nematicides, fungicides, and insecticides [20]. In Mexico, *T. erecta* is produced in large quantities every year for the celebration of “The Day of the Dead” in November. This also results in a lot of waste after the celebration. Nevertheless, the bioactive compounds present in *T. erecta* extracts make this species a potential reducing agent for AgNPs.

The applications of AgNPs are focused on different areas of biomedicine, biology, electronics, and agriculture, among others [21]. Currently, applications of AgNPs have become more prominent because of their antibacterial properties [22,23].

*Erwinia amylovora* is a native pathogen of wild rosaceous and is native to eastern North America. It is an important agronomic pathogen that shows antibiotic resistance [24]. This disease is called “fire blight” because it causes quick and serious plant injuries, and it is an important disease in apples (*Malus domestica*) and pears (*Pyrus communis*) in many parts of the world [25]. The “fire blight” bacteria attack buds and flowers, reducing significantly the production of pears and apples around the world. For this reason, *E. amylovora* has been classified as a quarantine organism in several countries [23,26,27].

Considering the phytochemical potential of *T. erecta* in the synthesis of AgNPs and the need for an antibacterial product to control “fire blight”, the present study explores the properties of leaf and petal extracts of *T. erecta* for the synthesis of long-lasting AgNPs with antibacterial properties to control *E. amylovora*, an important agronomic pathogen that shows antibiotic resistance. We evaluated the use of various volumes of leaf and petal extracts of *T. erecta* for the synthesis of AgNPs, identifying the principal functional groups responsible for synthesis and capping using thin-layer chromatography and Fourier transform infrared spectroscopy (FTIR). Once we determined the best synthesis method, we explored the particles’ characteristics in terms of longevity, morphology, and elemental composition. UV-Vis spectroscopy for surface plasmon resonance (SPR) was used to study their two-year longevity; transmission electron microscopy (TEM) was used for size and morphology; high-resolution TEM and selected area electron diffraction (SAED) for crystallinity; and X-ray energy-dispersive spectroscopy (EDS) for elemental composition. The antibacterial activity of the AgNPs against the plant pathogen, *E. amylovora*, was tested in vitro.

## 2. Results

### 2.1. AgNP Synthesis and UV-Vis Spectrophotometry

**Figure 1:**
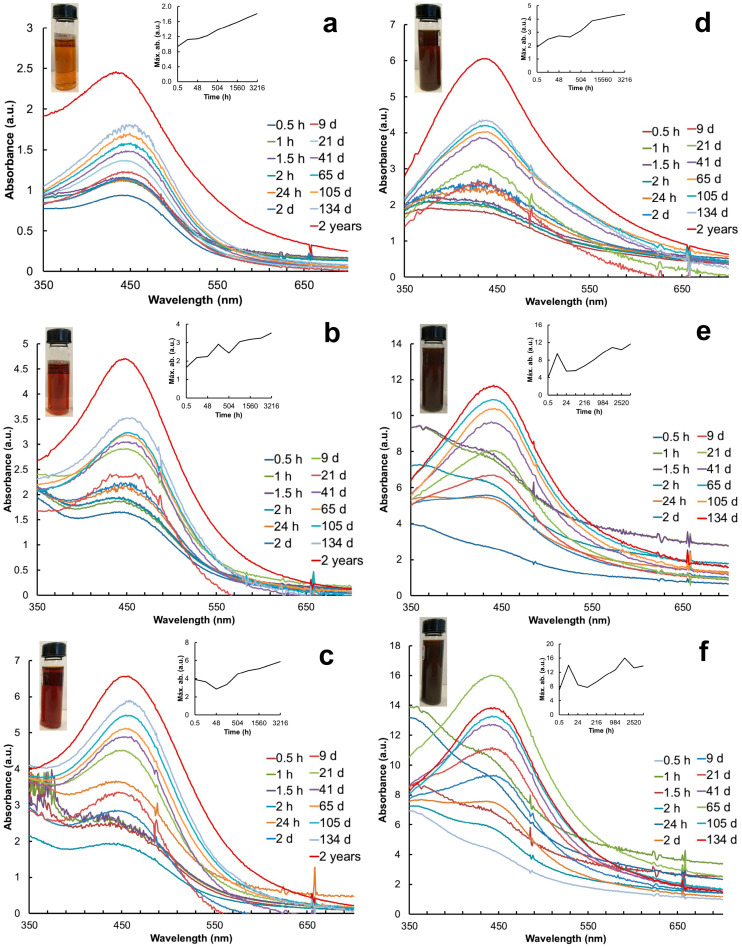
UV-Vis spectra of AgNPs synthesized with different volumes of aqueous extracts of leaves and petals of *T. erecta* for 2 years or until their precipitation. (**a**–**c**) Leaf extract; (**d**–**f**) petal extract; (**a**,**d**) 1 mL extract; (**b**,**e**) 3 mL extract; (**c**,**f**) 5 mL extract. Right insert corresponds to the stabilization of the system over time. Left insert shows colloid solutions of AgNPs. Color change is observed after 10 min of exposure to sunlight.

Within 2–3 min after mixing the plant extracts with the AgNO_3_ solution, the color changed from pale yellow to dark yellow, and after exposure to sunlight, the AgNP colloids changed to brown. The AgNPs synthesized with the leaf extract exhibited lighter brown colors than the ones synthesized with the petal extract (Figure 1). The pH of the colloids varied depending on the part of the plant used: the leaf extract produced colloids with a pH of 5.45–5.39; in contrast, the petal extract was more acidic (a pH of 3.94 to 3.48) depending on the volume used (Table 1).

Particle synthesis with the leaf extract revealed SPR at 30 min with the λ_max_ at 440 nm, 442 nm, and 404 nm with 1 mL, 3 mL, and 5 mL, respectively (Figure 1a–c). In contrast, particle synthesis with the petal extract showed SPR later, with the λ_max_ at 433 nm (3 mL) after 24 h and the λ_max_ at 447 nm (5 mL) after 2 days (Figure 1d–f). The AgNP colloids produced with 1 mL of leaf extract presented the most stable and consistent SPR spectra over time (Figure 1a). The absorbance (a.u.) of the SPR spectra increased as a function of time; the highest values were recorded with 5 mL of the petal extract (Figure 1f). The system stabilized after 21 days with the leaf extract and after 41 days with the petal extract. The SPR spectra of each treatment were monitored over time until precipitation. The AgNP colloids produced with the petal extract precipitated after 134 days (Figure 1e,f) whereas the ones produced with leaves showed SPR for up to 2 years and an increase in absorbance (Figure 1a–c). Table 1 shows the a.u. and λ of the AgNPs recorded before their precipitation. The Full Width at Half Maximum (FWHM) values indicated the high polydispersity of the AgNPs synthesized with 1 mL of the leaf extract in contrast to the petal extract (Table 1).

### 2.2. The Size and Sphericity of the AgNPs

**Figure 2:**
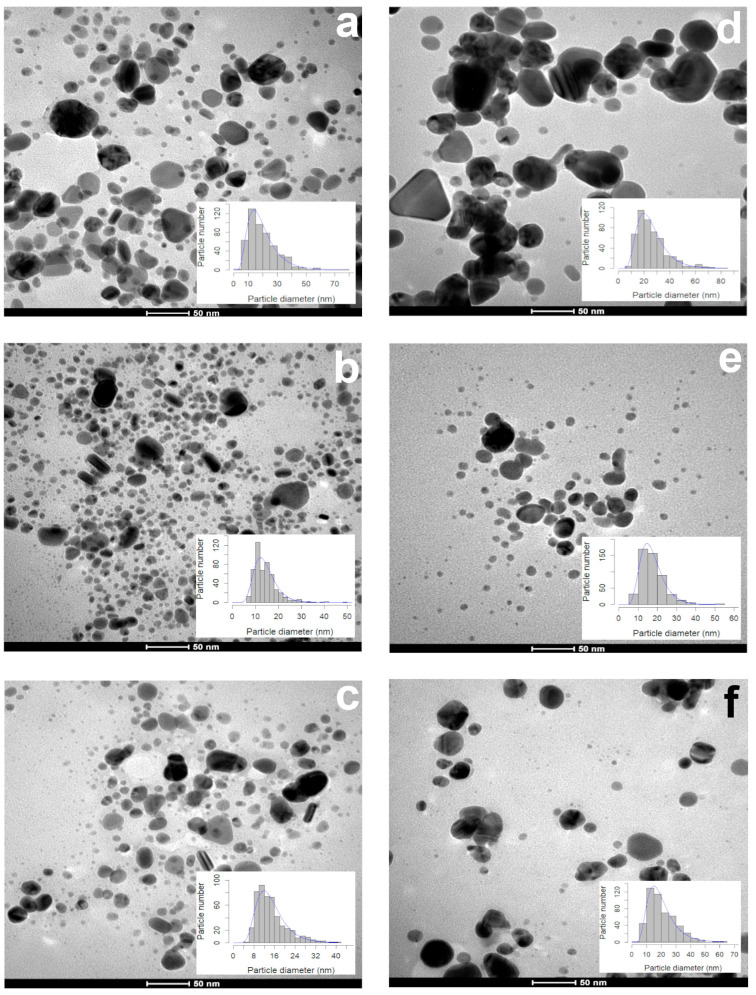
Morphology and diameter of AgNPs synthetized with different volumes of aqueous extracts of leaves and petals of *T. erecta*. TEM microphotographs and size distribution histograms: (**a**,**d**) 1 mL; (**b**,**e**) 3 mL; (**c**,**f**) 5 mL. Leaf extract (**a**–**c**) and petal extract (**d**–**f**). Data obtained 15 days after the synthesis (*n* = 500).

During the first 15 days, 1 mL of the leaf extract produced smaller (17.64 ± 8.87 nm) particles than the ones synthesized with the petal extract (23.05 ± 9.88 nm) (Figure 2a,d). The particle size (13.18–13.44 nm) and sphericity (0.85–0.86) obtained with 3 and 5 mL of the leaf extract were similar (Figure 2b,c). The volume used affected the particle size (3 mL, 16.45 ± 5.52 nm; 5 mL, 18.40 ± 9.41 nm (Figure 2e,f)), but the sphericity (0.86 ± 0.09) was not affected. According to the TEM photographs, the shape of all of the AgNPs synthesized with both extracts were quasi-spherical, showing a sphericity index of 0.85–0.86 (Table 1).

**Figure 3:**
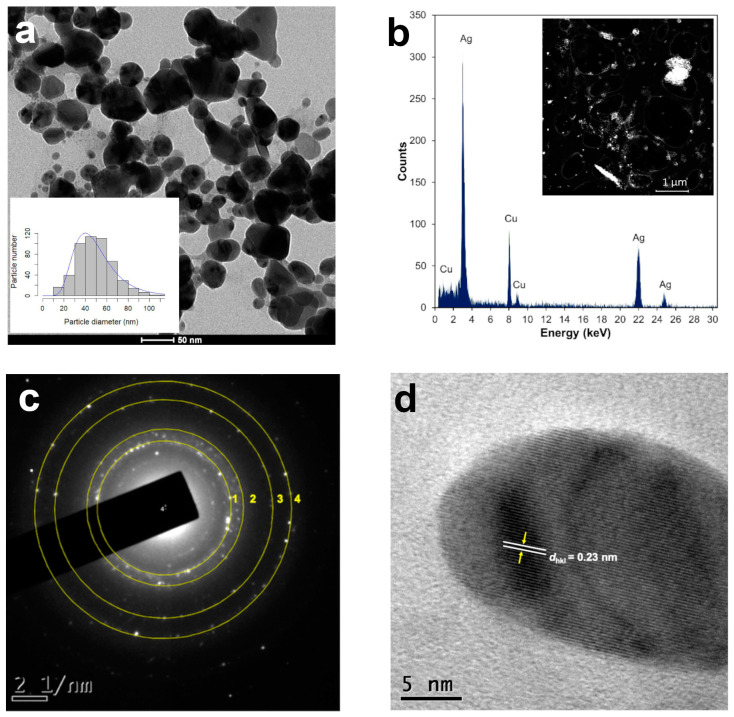
Structural analysis of AgNPs synthesized with 1 mL of *T. erecta* leaf extract: (**a**) TEM microphotographs and size distribution histograms (*n* = 500). (**b**) High-angle annular dark field (HAADF) imaging in STEM mode and X-ray energy-dispersive spectroscopy (EDS). (**c**) SAED pattern of single AgNPs exhibit four concentric rings, corresponding to face-centered cubic (fcc) structure, indicating polycrystalline Ag. (**d**) High-resolution TEM (HRTEM) image, yellow arrows indicate lattice fringes with a *d*_hkl_ value of 0.23 nm for AgNPs corresponding to the (111) plane. Data obtained 2 years after synthesis.

The diameter of the AgNPs produced with 1 mL of the leaf extract increased to 48.12 nm after two years, and the median sphericity decreased (0.82) (Figure 3a), indicating that the particles were larger and less spherical (Table 2), and most of them were seen to agglomerate (Figure 3a).

The Kruskal–Wallis test was performed to compare the medians of the diameters and the sphericity of the AgNPs produced with different volumes of the leaf and petal extracts.

### 2.3. Annular Dark Field (HAADF) in STEM Mode and EDS Elemental Microanalysis

Scanning transmission electron microscopy (STEM) is a combination of SEM and TEM techniques, whereby a beam of electrons is transmitted (TEM) through a thin sample, forming an atomic-resolution image. The STEM imaging mode has certain benefits compared with the broad-beam illumination mode; the main advantage is the use of a high-angle annular dark field (HAADF) detector, in which the images registered have different levels of contrast related to the chemical composition of the sample. The use of X-ray energy-dispersive spectroscopy (EDS) attached to a scanning electron microscope in STEM mode is a powerful technique for studying the chemical composition of NPs [28]. The HAADF-STEM images of the AgNPs synthetized using 1 mL of the *T. erecta* leaf extract are shown in Figure 3b. The EDS performed on the bright dots revealed the presence of characteristic peaks of silver (Ag) in the sample, confirming the elemental nature of the NPs (Figure 3b). The Cu signal in the spectra corresponded to the copper grids used to mount the AgNPs.

### 2.4. Selected Area Electron Diffraction Pattern and High-Resolution TEM

Selected area electron diffraction (SAED) is a crystallographic experimental technique typically performed using a transmission electron microscope (TEM) to determine structure. SAED is commonly used for phase identification, the determination of structural intergrowth, etc. SAED diffraction patterns are either simple spot patterns corresponding to single-crystal diffraction or ring patterns corresponding to powder diffraction from multiple crystals with a variable orientation. The SAED pattern of one of the spherical particles synthesized with 1 mL of the *T. erecta* leaf extract 2 years after synthesis indicates that they have a face-centered cubic (fcc) structure, indicating polycrystalline silver (Figure 3c). High-resolution TEM (HRTEM) is a powerful microscopic technique that uses very fine and highly energetic illumination, resulting in a high resolution that can structurally characterize samples at the atomic level. The HRTEM images obtained of the AgNPs produced with the *T. erecta* extracts clearly showed lattice fringes with spacing *d*_hkl_ values of 0.23 nm that in this case indicated a crystalline (111) plane, which reveals that the growth of Ag nanoparticles occurs preferentially on the (111) plane (Figure 3d). The clear lattice fringes in the HRTEM images and the typical SAED pattern with bright circular rings corresponding to the (111), (200), (220), and (311) planes show that the nanoparticles obtained are highly crystalline [29].

### 2.5. FTIR Spectral Analysis

**Figure 4:**
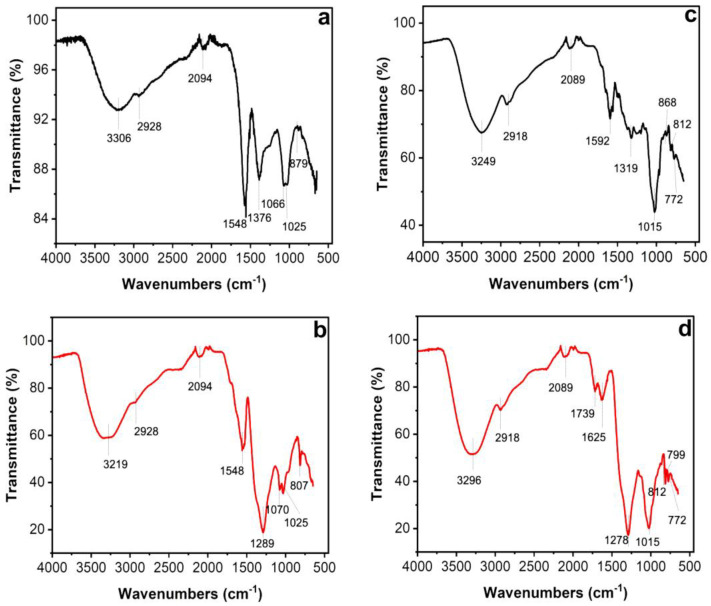
Fourier transform infrared spectroscopy (FTIR) spectra of leaves (**a**,**b**) and petals (**c**,**d**) of *T. erecta*. Black lines: FTIR spectra of aqueous extracts from leaves (**a**) and petals (**c**). Red lines: FTIR spectra of AgNPs synthesized using aqueous extracts from leaves (**b**) and petals (**d**) of *T. erecta*.

Fourier transform infrared spectroscopy, also known as FTIR analysis or FTIR spectroscopy, is an analytical technique that reveals the nature of the functional groups in an organic sample. The FTIR analysis method uses infrared light to scan test samples and observe their chemical properties [30]. Figure 4 shows the transmitted bands of the FTIR analysis that indicate the presence of functional groups that correspond to possible molecules involved in the biosynthesis and stabilization of the AgNPs. Both the leaf and petal extracts of *T. erecta* showed strong absorption bands at 3306 cm^−1^, 3219 cm^−1^, 3296 cm^−1^, and 3249 cm^−1^, corresponding to the stretching vibrations of the O-H groups of alcohols or phenols. The bands at 2928 cm^−1^ and 2918 cm^−1^ are attributed to N-H bonds, while the bands at 2094 cm^−1^ and 2089 cm^−1^ are attributed to C-H stretching vibrations. The bands at 1592 cm^−1^, 1625 cm^−1^, and 1548 cm^−1^ denoted C=O stretching vibrations. The intense bands present in both samples at 1376 cm^−1^, 1289 cm^−1^, and 1278 cm^−1^ indicated single C-O bonds. Stretching vibrations of the aromatic ring and phenyl groups were identified in the absorption bands at 879 cm^−1^, 868 cm^−1^, 807 cm^−1^, 812 cm^−1^, 799 cm^−1^, and 772 cm^−1^.

### 2.6. Qualitative Identification of Secondary Metabolites

**Figure 5:**
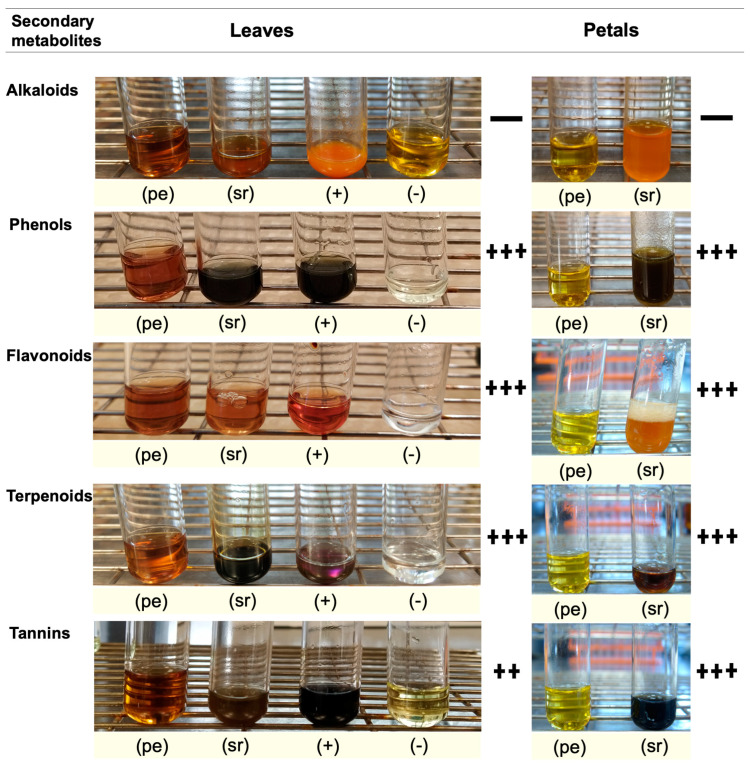
Preliminary analysis of secondary metabolites in aqueous leaf and petal extracts of *T. erecta*: pe, plant extract; sr, sample reaction; +, positive control; −, negative control; +++ very abundant; ++ moderately abundant; + slightly abundant; − not detected or absent. The abundance level of the secondary metabolite was determined qualitatively based on the positive (+) control.

**Figure 6:**
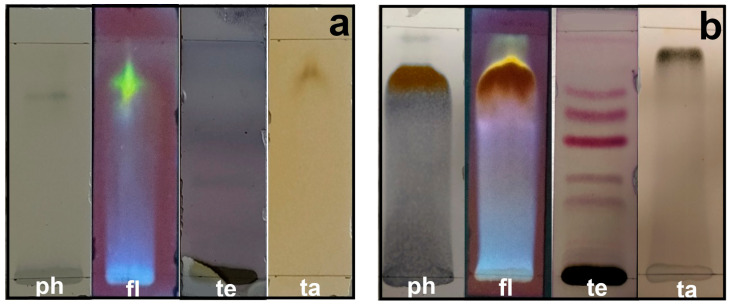
Thin-layer chromatography of methanol extracts of *T. erecta*: (**a**) leaves; (**b**) petals. ph, phenols; fl, flavonoids; te, terpenoids; ta, tannins.

Secondary metabolites are generally defined as small organic molecules produced by an organism but that are not essential to its growth, development, or reproduction. It has been reported that various secondary metabolites play a role in the synthesis of metallic nanoparticles [31]. The coloration technique, which implies changes in color, was used to identify secondary metabolites. The natural coloration of the extracts was yellowish for the petals and brown for the leaves. The secondary metabolites detected using this technique in the leaf and petal extracts were phenols, flavonoids, terpenoids, and tannins, but saponins and alkaloids were not detected (Figure 5). The presence of phenols was revealed by an intense blue coloration, while the existence of flavonoids was demonstrated by a color change to orange and effervescence (Figure 5). The presence of terpenoids and tannins was corroborated by pink-purple and dark blue coloration, respectively (Figure 5). Thin-layer chromatography confirmed the presence of compounds detected in the preliminary analysis (Figure 6). Terpenoids were abundant in the leaf and petal extracts, and phenolic compounds and tannins were more abundant in the petal extract than in the leaf extract. In the chromatography of the leaf extract, the lower band corresponded to high-polarity phenols, and the upper bands corresponded to nonpolar phenols (Figure 6). Bands corresponding to several types of flavonoids in the leaf and petal extracts were detected (Figure 6).

### 2.7. Antibacterial Activity of the Synthesized AgNPs

**Figure 7:**
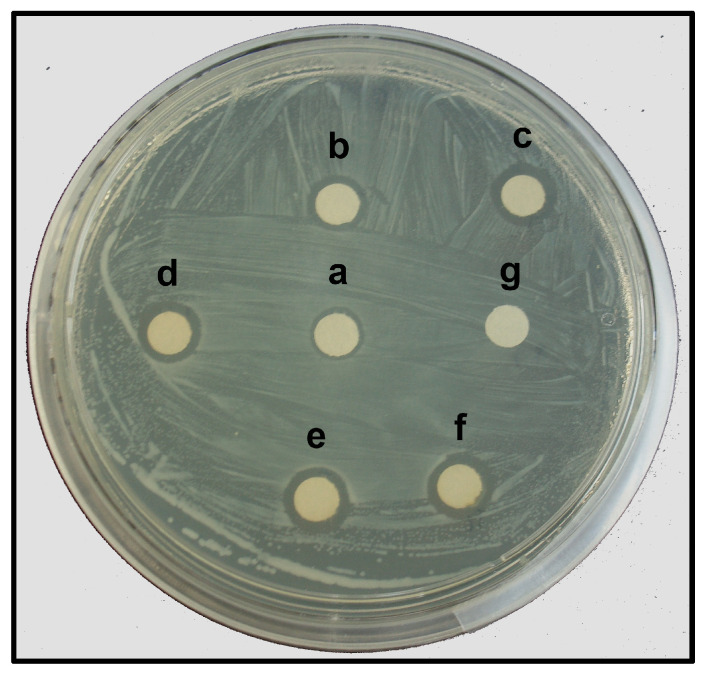
Antibacterial activity of AgNPs against *E. amylovora* according to Kirby–Bauer disk diffusion method. Concentration of AgNPs tested: a, 25 mg L^−1^; b, 50 mg L^−1^; c, 100 mg L^−1^; d, 200 mg L^−1^; e, 360 mg L^−1^; f, 360 mg L^−1^ of AgNO_3_ (positive control); g, deionized water (negative control).

The AgNPs synthesized with the leaf extract of *T. erecta* showed significant antibacterial activity against *E. amylovora*, indicated by the formation of inhibition zones (IZs) around the paper discs impregnated with the AgNPs (Figure 7). The largest IZ (9.88 mm diameter) was observed at the highest concentration (360 mg L^−1^) of the AgNPs. But this response was similar to the positive control, where AgNO_3_ was used at a similar concentration (Table 3). The diameter of the IZ of the paper discs with lower concentrations (100 mg L^−1^, 50 mg L^−1^, and 25 mg L^−1^) of the AgNPs was in function with the concentration. The IZ observed with 200 mg L^−1^ and 360 mg L^−1^ of the AgNPs was similar (Table 3); therefore, the lowest concentration with the highest inhibitory activity was 200 mg L^−1^ of the AgNPs.

## 3. Discussion

Several factors affect the reduction of Ag^+^ into AgNPs, such as sunlight, which is a pivotal factor in the rapid synthesis of AgNPs, as it can influence the shape of the AgNPs [32]. In green synthesis of AgNPs with extracts of *T. erecta*, sunlight played an important role as a photocatalyzer in the reduction of Ag^+^ ions into AgNPs because it accelerated the reduction, indicated by a change in color in 30 min, and the formation of typical SPR [5,33,34]. The SPR bands of the AgNPs of both the leaf and petal extracts were formed from 400 to 500 nm, as in other reports [35,36,37], but in our case, the SPR of the *T. erecta* AgNPs exhibited the typical spectra for up to 2 years. The structural properties of the AgNPs were corroborated using TEM and HRTEM. In our study, the term “long-lasting” refers to the AgNPs exhibiting SPR over a greater duration than the reported for other green synthesis. The literature mentions that the persistence of SPR over time, independent of the plant organ used, is an indication of the continuous synthesis of NPs [3,12,38], and the stability of the AgNPs is indicated by the unchanged λ_max_ values of the SPR [39,40,41]. It is important to mention that although the synthesis method with 1 mL of the leaf extract of *T. erecta* produced long-lasting (2 years) AgNPs, the λ_max_ of the SPR shifted from 433 nm to 440 nm, and the size increased from 17.64 nm to 48.12, but the shapes did not vary significantly (0.85–0.86 sphericity index). Similar trends were observed with other plant extracts: *Cornus officinalis* plants [38]; the leaves of *Clitoria ternetea* and *Solanum nigrum* [42]; the leaves of *Calliandra haematocephala* [34]; *Artemisia dracunculus* plants [43]; and the leaves of *Leucas aspera* [44]. Some variables that can influence the size of NPs are the concentration of the reducing agent, the plant species, the part of the plant used, and pH [7,38,45].

In our study, we observed a positive relationship between the absorbance (a.u.) and the plant extract volume: the lower the concentration of the extract, the lower the absorbance values (a.u.). This suggests a low nucleation rate and fewer particles of a large size [46].

For the reduction and “capping” of the AgNPs, it is possible that the molecules present in the leaf extract were more active than those in the petal extract, explaining the durability of the AgNPs produced with the leaf extract, as has been reported for other species [47,48]. According to Huang et al. [49] and Kulkarni and Muddapur, [50], the reduction of Ag^+^ into AgNPs and their stabilization is facilitated by secondary metabolites (alkaloids, tannins, phenols, saponins, and terpenoids) and other molecules such as proteins, amino acids, enzymes, polysaccharides, and vitamins. The FTIR analysis clearly indicated the participation of phenolic compounds found in the leaf and petal extracts of *T. erecta* during the synthesis of the AgNPs. The literature suggests that phenolic compounds contain O-H and C-O groups that have a strong tendency to bind metal ions, playing a crucial role in the nucleation and stabilization of the nanoparticles [31,51]. Maji et al. [52] and Burlec et al. [53] reported that flavonoids interact with metal ions through carbonyl functional groups, releasing reactive hydrogen, which converts the enol into a keto-like flavonoid, leading to the formation of Ag^+^. Additionally, the functional groups C=O of the carboxyl and C-H of the aromatic compounds identified in *T. erecta* could also play a crucial role in the reduction and stability of the AgNPs, as was observed during the functionalization of silver nanoparticles [30].

The diffraction patterns and interplanar distances observed in the AgNPs synthesized with 1 mL of the *T. erecta* leaf extract after two years of synthesis confirmed their crystalline nature (fcc), as reported for the AgNPs [54,55,56,57]. To our knowledge, this is the first study that monitors the SPR trends in the particles over 2 years. The SPR trends could be used as a preliminary approach to monitoring the size and shape of long-lasting NPs before analyzing them with more specific techniques.

This is the first research that explores the antibacterial activity of AgNPs against *E. amylovora* bacteria. This pathogen is a Gram-negative bacterium, the causal agent of fire blight, and it is considered a devastating pathogen for the Rosaceae family worldwide [58]. The current control methods for this pathogen involve the use of chemical compounds or antibiotics that become ineffective due to the natural evolution of bacterial resistance to these agents [59]. The use of AgNPs against bacteria showing antibiotic resistance is attracting the attention of researchers. Our results indicated that AgNPs (17.64 nm) synthesized with the leaves of *T. erecta* showed a bactericidal effect against *E. amylovora* as a function of the concentration used. Several hypotheses have been proposed to explain the antibacterial mechanisms: the direct interaction of the AgNPs with the cell membrane of the bacteria, causing irreversible damages; the interaction of the AgNPs with the -SH (thiol) groups; and the production of reactive oxygen species (ROS) and the release of silver ions that inhibit the respiratory enzymes in the mitochondria, which also generates ROS [41].

The toxicity of AgNPs to *E. amylovora* may be related to the release of Ag^+^ ions that interact with the bacteria cell wall and membrane, creating pores, affecting the membrane permeability, releasing cytoplasmic contents, and ultimately causing cell death [13,22,60,61]. The particle size can also influence the antibacterial activity, with smaller particles exhibiting a greater toxicity than larger AgNPs [62]. These effects could be facilitated by the thin cell wall structure of Gram-negative bacteria, composed of a single layer of peptidoglycan [63].

According to Xu et al. [64], a particle size of up to 80 nm is considered small enough to penetrate the inner and outer barriers of bacteria. The size of the AgNPs produced and tested in the present research were smaller (17.64 nm) and therefore capable of crossing and perturbing the bacteria cell wall and cell membrane. Additionally, the phenols present in the plant extracts of *T. erecta* could produce scavenging agents with the capacity to generate oxidative stress in the bacteria. We believe that the antibacterial capacity of the AgNPs produced in the present research is a result of the synergic action between the phenols and the Ag^+^ ions. Biological control of “fire blight” using AgNPs, synthesized using the presented protocol, offers a novel and ecofriendly alternative for preventing or controlling *E. amylovora*.

## 4. Materials and Methods

### 4.1. Plant Material

The floral stems of *T. erecta* were acquired from the Experimental Field “San Ignacio” at the Chapingo Autonomous University (Universidad Autónoma Chapingo), Texcoco, the State of Mexico. The leaves and petals were separated, washed with deionized (DW) water, and dried in an oven at 40 °C for 48 h until a constant weight was obtained. The dried biomass was grounded and stored in paper bags in the dark at 20 °C until use.

### 4.2. Silver Nanoparticle Synthesis

The aqueous extracts were prepared by adding 1 g of the powdered leaves or petals to 100 mL of boiling (95 °C) DW for 10 min. The extract obtained was cooled to 25 °C and filtered (Whatman^®^ No. 40, Maidstone, Kent, UK) with a vacuum and then centrifuged (Hettich centrifuge, Tuttlingen, Germany, EBA 20, 2002-01) at 1180 g for 10 min. Then, the extract was stored in an amber glass bottle at 4 °C before use.

**Figure 8:**
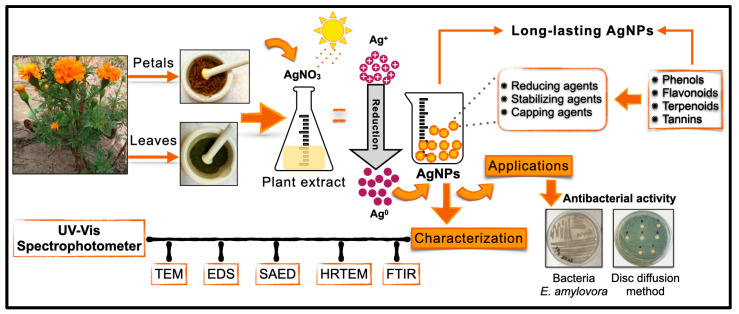
Schematic illustration of the green synthesis of AgNPs assisted with leaf and petal extracts of *T. erecta*, characterization, and antibacterial activity against *E. amylovora*.

Different volumes (1, 3 and 5 mL) of the leaf and petal extracts of *T. erecta* were added to 5 mL of an AgNO_3_ (10 mM) solution (Grade ACS reagent, ≥99.0%, Sigma-Aldrich, St. Louis, MO, USA) and gauged to 15 mL with DW. The vial with the mixture was exposed to sunlight for 10 min until the color of the solution changed. The pH was recorded, and the AgNPs were stored in darkness at 4 °C. The treatments were different volumes (1, 3, and 5 mL) of the leaf and petal extracts, making a total of 6 treatments for AgNP synthesis (Figure 8).

### 4.3. UV-Vis Surface Plasmon Resonance (SPR)

The SPR of the AgNPs was analyzed using a UV-Vis spectrophotometer (HP 8453 UV-Visible Spectroscopy Systems, Waldbronn, Germany) in the 350 to 700 nm range at a resolution of 1 nm. For each treatment, the AgNP solutions were monitored every 30 min, for the first 2 h, then every 24 h, then every 7 d, every month until 134 d, and finally at 2 years and/or until the presence of precipitates. The Full Width at Half Maximum (FWHM) values were obtained from the SPR using OriginPro software (version number: 9.6.5.27, 2018) [65].

### 4.4. The Morphology, Size, and Structure of the AgNPs

Studying the shape and size of the AgNPs was carried out using a Tecnai G2 Spirit Twin transmission electron microscope (TEM) (Thermo Fisher, Waltham, MA, USA). A volume of 5 μL of the diluted aqueous solutions (1:10) of the NPs was mounted onto copper grids (Mesh 200) covered with Formvar and carbon (Ted Pella, Inc. Redding, CA, USA) A total of 500 NPs from 15 microphotographs were analyzed per treatment; the diameter and sphericity index were calculated using Image J software (Version number: ImageJ 1.53k/java 1.8.0_172) [66], and a size distribution histogram was elaborated using the R statistical package [67]. The medians (Me) and the median absolute deviation (±MAD) were calculated for the AgNPs’ diameters and sphericity. Because the data were not normally distributed, we performed the non-parametric Kruskal–Wallis test. When the null hypothesis for the Kruskal–Wallis test was rejected (α = 0.05), then a pairwise comparison between groups was performed using the “pairwise.wilcox.test”; this function is available in the stats package in R with Holm correction [68] for multiple comparisons. The grouping of treatments was performed using the function “orderPValue” available in the “agricolae” package in R using the results from the “pairwise.wilcox.test” function. We also set α = 0.05. The microphotographs were taken 15 days after synthesis and 2 years after synthesis of the AgNPs synthesized with 1 mL of the *T. erecta* leaf extract.

Structural characterization of the AgNPs was performed using a JEM-2100 JEOL high-resolution transmission electron microscope operated at 200 kV, and the crystalline pattern of the AgNPs was analyzed according to the selected area electron diffraction (SAED) patterns. The specimens were prepared on an ultrathin carbon film on Lacey (400-mesh) copper grids. To evaluate the interplanar distance d (d-spacing), a fast Fourier transform (FFT) was performed using DigitalMicrograph software (version number: 3.5) [69], and Image J software (version number: ImageJ 1.53k/java 1.8.0_172) [66] was used to index and identify the crystallographic planes. This characterization was performed 2 years after synthesis on the AgNPs synthesized with 1 mL of the aqueous extract of the *T. erecta* leaves.

### 4.5. High-Angle Annular Dark-Field Scanning Transmission Electron Microscopy (HAADF-STEM) and Energy-Dispersive X-ray Spectroscopy (EDS)

Aqueous colloids of the AgNPs were diluted (1:10) with DW and sonicated twice for 15 min. An aliquot of 5 μL was mounted onto an ultrathin carbon film supported by a Lacey (400-mesh) copper grid and examined using HRTEM (Tecnai F30, Fei Company, Hillsboro, OR, USA), operating at 300 kV. Micro-elemental analysis of the NPs was performed using energy-dispersive spectroscopy (EDS), along with “high-angle annular dark-field in scanning transmission electron microscopy” (HAADF-STEM). This technique is highly sensitive to variations in the atomic number of atoms in the sample (Z-contrast images), and the EDS analysis can be more easily directed to the particles.

### 4.6. Fourier Transform Infrared Spectroscopy (FTIR)

To investigate the functional groups that participate in the reduction of silver ions, FTIR was used. A volume of 15 mL of the AgNPs was lyophilized in a freeze dryer (Labconco FreeZone 4.5 L Benchtop Freeze Dry System, Kansas City, MO, USA) for 24 h with three repetitions per treatment. The infrared spectra were recorded using an Agilent Cary 630 FTIR spectrometer, Santa Clara, CA, USA. from 4000 to 400 cm^−1^ with a resolution of 1 cm^−1^. This analysis was performed on an aqueous extract before and after the AgNP synthesis. The results were plotted using OriginPro software (version number: 9.6.5.27, 2018) [65].

### 4.7. Preliminary Test for the Secondary Metabolites

The families of the secondary metabolites were analyzed: saponins (aqueous extracts) and phenols, tannins, flavonoids, alkaloids, and terpenoids (methanol extracts). A positive control and a negative control were used in all the tests. For the aqueous extracts, 1 g of dried and grinded plant material was added to 30 mL of deionized water at 22 °C and subjected to an ultrasonic bath (Emerson Bransonic M1800, Mex, Mexico) 70 W at 22 °C for two periods of 10 min each; then, the extract was centrifuged (Hettich centrifuge, EBA 20, 2002-01, Tuttlingen, Germany) at 1180 g for 10 min, and the supernatant was recovered. For the methanolic extracts, 1 g of dry and grinded biomass was added to 10 mL of 80% methanol, and a similar procedure was followed to the one for the aqueous extracts. Subsequently, 0.5 mL of the aqueous or methanolic extracts was placed in test tubes for preliminary analysis of the secondary metabolites following the methodology reported by Soto-Hernández et al. [70].

### 4.8. Thin-Layer Chromatography

Thin-layer chromatography was conducted based on the preliminary analysis of the secondary metabolites. The methanol extracts were concentrated in beakers of 5 mL. The elution media of ethyl acetate–methanol (1:1, *v*/*v*) was used for the flavonoids (9:1, *v*/*v*), tannins, and phenols. The elution media hexane–ethyl acetate (8:2, *v*/*v*) was employed for the terpenoids. The extracts were applied to glass plates of 2.5 × 7.5 cm (Macherey-Nagel^TM^ 821005, Adamant TLC, UV254, Düren, Germany), and the position of the sample was corroborated under an UV light Darkroom Chromatography Viewer (Cole-Parmer 9818 Series, Chicago, IL, USA). Then, chromatography was carried out. Finally, the plates were revealed with Natural Product/Polyethylene Glycol (NP-PEG) for the flavonoids, Folin–Ciocalteu reagent for the phenols, FeCl_3_ for the tannins, and vanillin-H_2_SO_4_ (1% in ethanol) for the terpenoids, according to Soto-Hernández’s methodology [70].

### 4.9. Antibacterial Activity of the Silver Nanoparticles

The antibacterial activity of the AgNPs synthesized with 1 mL of the aqueous extract of the *T. erecta* leaves against *E. amylovora* was evaluated using the standard Kirby–Bauer disk diffusion method [22,71,72,73]. Sterile paper discs (Whatman No. 1) of a 6 mm diameter were soaked in five concentrations of the AgNPs (25 mg L^−1^, 50 mg L^−1^, 100 mg L^−1^, 200 mg L^−1^, and 360 mg L^−1^), and one positive control (360 mg L^−1^ AgNO_3_, 10 mM) and one negative control (DW) were used, making a total of seven treatments. The soaked discs were air-dried under sterile conditions until use. Pure cultures of the bacterium were cultivated at 1.5 × 10^8^ CFU/mL (according to the McFarland scale) on standard agar plates (23.5 g of dehydrated culture medium in 1 L of DW) (Bioxon, Becton Dickinson, Mexico City, Mexico, S. A. de C. V.) using a sterile inoculating “Drigalsk” loop. Then, 7 discs (one from each treatment) were placed in each of the six Petri dishes (repetitions) under sterile conditions using a laminar flow hood (Labconco, 302411100, Kansas City, KS, USA). The plates were incubated at 28 ± 2 °C for 24 h. The diameter (mm) of the inhibition zone (IZ) of bacterial growth around each disc was measured. Three independent experiments were conducted; there were 42 observations recorded for each experiment, making a total of 126 observations. The data were subjected to an analysis of variance (ANOVA), and the means were compared using Tukey’s test with an α = 0.05. The R statistical package was used for the analyses [67].

## 5. Conclusions

This is the first report of the green synthesis of long-lasting (2 years) AgNPs assisted with *T. erecta* extracts. The SPR of the particles was monitored using UV-Vis spectroscopy, the morphology and size was characterized TEM and the elemental composition, and crystallinity were tracked using TEM-EDS, HRTEM and SAED.

The ideal conditions for the maximum synthesis of long-lasting AgNPs were found to be a leaf extract volume of 1 mL, with which the AgNPs were quasi-spherical (0.86) and had a diameter of 17.64 nm, a λ_max_ value at 440 nm, a polycrystalline fcc structure (111) corresponding to Ag^0^, and an interplanar distance of 0.23 nm. When they are maintained in the dark at 4 °C, they last two years, but their size increases to 48.12 nm and the λ_max_ value moves to 433 nm. However, the sphericity index (0.82) did not significantly change. These characteristics are important to consider for possible future technological applications.

The molecules detected in the extracts responsible for the synthesis included phenols, flavonoids, and terpenoids, in both the leaf and petal extracts. However, the metabolites in the leaf extract exhibited greater efficiency as reducing and capping agents compared to those in the petals. A statistical contribution of the present report is the proposal of using median (Me) data with the absolute deviation of the median (±MAD) instead of the mean and standard deviation to describe the diameter and sphericity of the particles because they better describe the particle population and its variation as a group in statistical terms.

This is the first report on the antibacterial activity of AgNPs against *E. amylovora*, one of the most threatening diseases in apple and pear plantations.

## Figures and Tables

**Table 1:** Attributes of AgNPs synthesized with leaf and petal extracts of *T. erecta*: pH, absorbance, wavelength, and statistical median comparison of diameter and sphericity of AgNPs between treatments (extract volumes).

Extract Volume (mL)		pH	Absorbance * (a.u.)	Wavelength * (nm)	Particle Size (nm)	FWHM (nm)	Particle Sphericity Index
Leaves	1	5.45	2.46	433	17.64 ± 8.87 a	188	0.85 ± 0.09 b
	3	5.39	4.70	448	13.18 ± 4.00 b	160	0.86 ± 0.07 a
	5	5.39	6.56	456	13.44 ± 5.03 b	175	0.85 ± 0.09 b
Petals	1	3.94	6.0	443	23.04 ± 9.88 a	158	0.86 ± 0.09 NS
	3	3.64	11.69	441	16.45 ± 5.52 c	148	0.86 ± 0.07 NS
	5	3.48	13.79	448	18.40 ± 9.41 b	156	0.85 ± 0.08 NS

The values are the median ± MAD (*n* = 500). Different letters indicate significant differences (pairwise Wilcoxon sum rank test, *p* < 0.05) between the extract volumes. NS = not significantly different (*p* < 0.05). * The absorbance and wavelength data are the maximum values over time recorded in the UV-Vis spectra. FWHM, Full Width at Half Maximum.

**Table 2:** Effect of time on the diameter and sphericity of AgNPs of *T. erecta* (1 mL).

AgNPs	Particle Size (nm)	Particle Sphericity
15 days after the synthesis	17.64 ± 8.87 a	0.85 ± 0.09 a
2 years after the synthesis	48.12 ± 17.01 b	0.82 ± 0.10 b

The values are the median ± MAD (*n* = 500). Different letters indicate significant differences (pairwise Wilcoxon sum rank test, *p* < 0.05).

**Table 3:** Antibacterial activity of AgNPs against *E. amylovora* measured as the inhibition zone (IZ) produced by paper discs impregnated with different concentrations of AgNPs synthesized with 1 mL of *T. erecta* leaf extract.

Concentration of AgNPs (mg L^−1^)	IZ (mm)
25	7.66 ± 0.29 d
50	8.56 ± 0.53 c
100	9.12 ± 0.40 b
200	9.56 ± 0.48 ab
360	9.88 ± 0.60 a
Positive control (360 mg L^−1^ of AgNO_3_)	9.91 ± 0.41 a
Negative control (Deionized H_2_O)	NA

Data are means ± SD of three independent data sets. Different letters indicate significant differences (Tukey’s test, *p* < 0.05). NA, no activity. The paper disc’s diameter was 6 mm.

## Data Availability

The data will be made available upon request.
